# Slow integration leads to persistent action potential firing in distal axons of coupled interneurons

**DOI:** 10.1186/1471-2202-12-S1-O17

**Published:** 2011-07-18

**Authors:** Mark EJ Sheffield, Tyler K Best, Brett D Mensh, William L Kath, Nelson Spruston

**Affiliations:** 1 Department of Neurobiology and Physiology, Northwestern University, Evanston, Illinois 60208, USA; 2 Department of Physical Medicine and Rehabilitation, Harvard Medical School, Boston, Massachusetts, USA; 3 Department of Applied Mathematics, Northwestern University, Evanston, Illinois 60208, USA

## 

The conventional view of neurons is that synaptic inputs are integrated on a timescale of milliseconds to seconds in the dendrites, with action potential initiation occurring in the axon initial segment. In a subset of rodent hippocampal and neocortical interneurons in acute slices prepared from serotonin 5b receptor (Htr5b) BAC transgenic mice [1], we found a much slower form of integration leading to action potential initiation in the distal axon. In approximately 80% of these interneurons (n=214 of 274), and in 23% of hippocampal interneurons in wild-type C57BL/6 mice (n=6 of 26), hundreds of spikes, evoked over a period of minutes, resulted in persistent firing that lasted for a similar duration.

Persistent firing was observed in response to step current injections, synaptic stimulation, sine wave current injections or in response to stimulation with natural spike trains [2]. With all of these protocols, multiple stimuli were required to induce persistent firing. While axonal action potential firing was required to trigger persistent firing, somatic depolarization was not; antidromic stimulation of the axon while hyperpolarizing the soma with current injection produced persistent firing. In addition, phase plots of persistent firing revealed that spikes had two components: an initial component represented spiking in the axon and a second component that overlapped with the current-evoked spikes, indicative of a somato-dendritic spike following an initial, axonally initiated spike.

In some recordings (n = 11), partial spikes (spikelets) were observed during persistent firing. These spikelets overlapped the first component of the full-amplitude spikes, with the peak of the spikelets corresponding to an inflection on the rising phase seen in the full-amplitude spikes. These observations suggest that the first component of each action potential during persistent firing is an axonal spike, which sometimes fails to evoke a somato-dendritic spike. Furthermore, in some cells (n=3), spikelets were observed if the soma was hyperpolarized during persistent firing. These spikelets were smaller than those observed without hyperpolarization, suggesting that they are caused by propagation failures at a more distal axonal location.

Using a stylized computational model constructed with the NEURON simulation environment [3] of a branching axon attached to a soma, we simulated both small- and large amplitude spikelets, as well as full-amplitude spikes, by depolarizing a branch of the axon during somatic hyperpolarization. Large-amplitude spikelets corresponded to failure of the action potential to invade the soma, whereas small-amplitude spikelets corresponded to failures at the axon branches, 40 μm from the soma. Similar results were obtained with a full morphological model of a branching axonal arborization.

Additionally, in paired recordings, persistent firing was not restricted to the stimulated neuron; it could also be produced in the unstimulated cell (n=3). None of these pairs exhibited direct electrical coupling, and both glutamate and GABA receptors were blocked.

Consolidating these results suggests the existence of a previously unknown operational mode for some mammalian neurons. These interneurons can slowly integrate spiking, share the output across a coupled network of axons and respond with persistent firing even in the absence of input to the soma or dendrites.
